# Study on Lubrication Characteristics of Novel Forced Wave Generator of Harmonic Drive without Flexible Bearing

**DOI:** 10.3390/ma15010215

**Published:** 2021-12-28

**Authors:** Hongli Jia, Hongbing Xin

**Affiliations:** School of Artificial Intelligence, Beijing Technology and Business University, Beijing 100048, China; jiahli101@163.com

**Keywords:** harmonic drive, flexible bearing, elliptical sliding bearing, hydrodynamic lubrication, computational fluid dynamics (CFD)

## Abstract

In contrast to the conventional forced wave generator which consists of cam and flexible bearing in harmonic drive, the novel forced wave generator retains cam but cancels flexible bearing. In this article, the lubrication characteristics of the novel forced wave generator in harmonic drive is studied. First, an elliptical sliding bearing (ESB) model of simplified structure between the novel forced wave generator and the flex spline is established. Further, the computational fluid dynamics (CFD) method is employed to study the effect of some factors on the lubrication characteristics of the ESB model including elliptical gap ratio, width, and rotational speed. According to the analysis, the elliptical gap ratio has a great impact and its optimal value is 3, which is used in the design of the novel forced wave generator. Last, the practical design of the novel forced wave generator in harmonic drive is given, which can provide a basis for design and optimization of a forced wave generator without flexible bearing of the harmonic drive.

## 1. Introduction

In the 1950s, the harmonic drive (HD) was invented by C.W. Musser based on the theory of the thin-walled elastic deformation [1], which has a wide application owing to its significant advantages such as huge reduction ratio and extremely compact structure.

A conventional HD consists of three major components of wave generator (WG), flex spline (FS), and circular spline (CS) and the forced wave generator (FWG) is a common type of wave generator, which is an assembly of a flexible bearing fitted onto the contour of a cam. The role of the flexible bearing is to transmit deformation and reduce friction between the cam and FS. Harmonic drive is a kind of high-precision reducer by means of the periodic controllable elastic deformation generated by the FS and WG to transmit the motion and power. As we know, the design parameters of the WG profile directly affect the shape of the FS, and the working stress and the service life of the FS greatly determine the transmission performance of the whole harmonic reducer. In order to improve the meshing performance and extend the service life of harmonic gear transmission, Gravagno [2] discussed and quantitatively evaluated the influence of the wave generator shape on the pure kinematic error and centrodes of harmonic drive. Routh [3] analyzed the coning phenomenon and the lubrication mechanism at flex spline cup and cam interface in conventional harmonic drives using FEM method. Ma [4] investigated the friction behavior of the harmonic drive at low speed using experimental and numerical methods. Xin [5] set up the analysis model of triple wave type harmonic drive and worked out the FS’s radial and tangential deformation expressions under the action of triple disk type wave generator by using the elasticity theory. Li [6] proposed the wave generator profile for harmonic drive using a closed convex curve generated by the support function and analyzed the stress and deformation using FEM method. Bhabani [7] used the finite element method and experiments to compare and analyze the stress and strain of the flex spline in a conventional cam wave generator and a split cam wave generator without assembling the ring gear. Deformation stress of a flex spline under the action of a wave generator directly affects the service life of the flex spline and meshing quality of the meshing pair. Xin [8] developed the double-circular-arc tooth profiles of basic rack of harmonic drive with elliptical wave generator, which ensured the tooth profiles of flex spline and rigid spline to keep on conjugating with each other during the meshing period. Wang [9] proposed a new deformation model for a flex spline and established the mathematical model, which incorporates forced deformation in the working area and free deformation in the non-working area. The model is developed and analyzed in FEM. Ostapski [10] presented the problem of failure of the elastic bearing supporting the generator in a harmonic drive and a solution to the problem of elastic deformation of thin-walled shell structures with complex shapes within the theory of geometrically non-linear shells. Majchrak [11] proposed preparation of the finite element 3D computational model of the harmonic drive gear and tooth backlash analysis. Hrcek [12] performed a global sensitivity analysis of various design parameters affecting the lost motion of the harmonic drive and analyzed in FEM.

Finally, this paper establishes the model of the novel forced wave generator without flexible bearing contrary to the conventional forced wave generator which consists of cam and flexible bearing and uses the numerical method to improve the lubrication characteristics of the novel forced wave generator in the HD.

For the traditional HD, the application of the FWG with flexible bearing has problems. Due to the limitation of the principle of HD and the manufacturing process of flexible bearing, the minimum diameter of flexible bearing has a limitation, which is adverse to further development of HD including micro HD. Moreover, the life of flexible bearing determines the service life of the HD device, resulting in high demand of the design and manufacture of flexible bearing along with cost increase. In addition, on some special occasions, there is a demand for FWG without flexible bearing of HD. Examples include in the extreme transmission of the application of short service life HD [13]; occasions that require a larger hollow inner diameter of FWG to accommodate the motor and cables of the application of hollow-style HD [14]; and occasion such as small manipulator joints with extremely restricted space of highly demand of further compact structure of HD [15,16]. The novel FWG, that is the forced wave generator without flexible bearing named as the novel forced wave generator (NFWG), of HD appears as an important and promising technical means.

## 2. Materials and Methods

**Figure 1:**
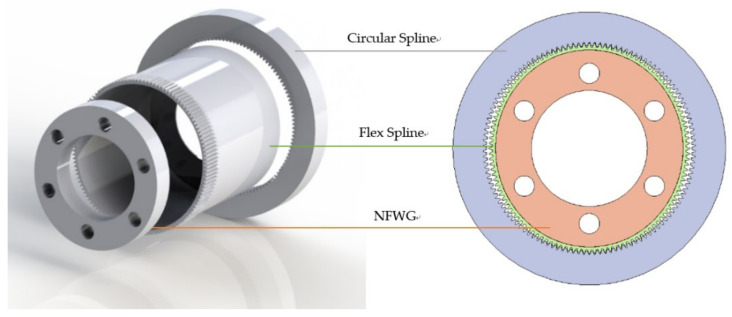
Components and geometry of the HD with NFWG.

As shown in Figure 1, the HD with NFWG also includes three key components, but the wave generator is NFWG. The NFWG is inserted into the FS from its open end, and the closed end of the FS is connected with the output shaft or housing. Normally, the CS is fixed and the NFWG and the FS work as the input and output parts, respectively. The gear engagement of the HD is realized through the elastic deformation of the FS. When the NFWG is driven by a motor and rotates in the FS, the FS produces flowing deformation wave and meshes with the CS, and an opposite micro rotation relative to the NFWG is achieved at the output end of FS. The FS is generally fabricated by high-strength alloy steel for aviation with good toughness, and the CS and the NFWG are generally by alloy steel or structural steel. As shown in Figure 1, for the HD without flexible bearing, the FS will be severely worn if no measures are taken.

If sufficient lubrication conditions can be ensured so that the NFWG and the inner wall of the FS can generate hydrodynamic lubrication, which can greatly reduce friction loss and surface wear, this would increase the life of the HD with the NFWG. Therefore, to improve lubrication performance and optimize lubrication characteristics between the NFWG and the FS in the HD, the analysis is carried out with CFD simulation in ANSYS FLUENT environment.

The factors that affect the lubrication performance between the NFWG and the FS include the size parameters of the structure, the gap between the NFWG and the FS, and the operating parameters such as the rotational speed of the NFWG and the lubricant. For this research, in the case of a given lubricant and harmonic drive unit, that is, the inner diameter of the FS, the influence of the design parameters of the NFWG including the width of the NFWG, the gap ratio between the NFWG and the FS, and the rotational speed of the NFWG on the lubrication characteristics of the model is simulated and analyzed.

## 3. Modeling

### 3.1. Mathematical Model

**Figure 2:**
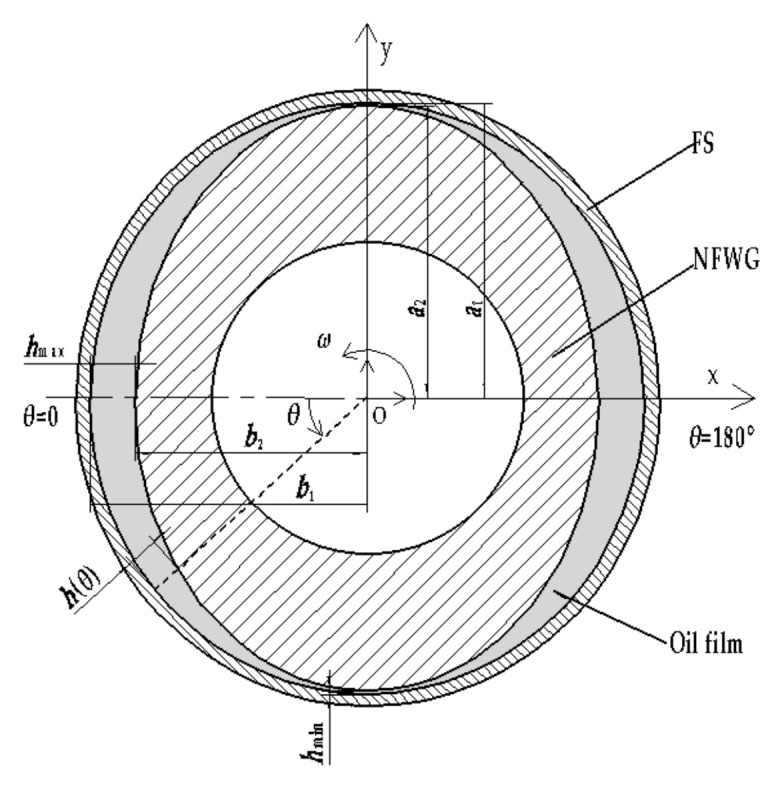
The ESB model of the simplified structure between the NFWG and FS.

As shown in Figure 2, an ESB model for CFD analysis which is an elliptical sliding bearing (ESB) model of the simplified structure between the NFWG and FS was established. The center of the NFWG and the FS is represented by O and the coordinate system is given. The NFWG rotates with a rotational speed *n*_1_, and the FS rotates slightly based on transmission ratio in the opposite direction to the NFWG. The outer wall of the NFWG and the inner wall of the FS are boundaries of the oil film. The oil film thickness *h*(*θ*) varies from the maximum value *h*_max_ at rotation angle *θ* = 0 to the minimum value *h*_min_ at *θ* = 90°. The width direction of the NFWG is the z-axis, and the half-width position is set to *z* = 0. The region between the outer wall of the NFWG and the inner wall of the FS is occupied by a lubricant. A rigid aligned ESB model with the geometry of Figure 2 is considered.

The parameters of the model are as follows: *a*_1_ = 25 mm, *b*_1_ = 24.7 mm, *a*_2_ = 24.9 mm, *r* = 24.85 mm, *b_w_* = 20 mm, *L* = 20 mm, and the range of *b*_2_ is 23.5 mm to 24.6 mm.

The elliptical sliding bearing (ESB) model proposed herein is different from the ordinary non-circular sliding bearing model. The two parts constituting the ESB are both elliptical whereas one part of the ordinary non-circular sliding bearing is circular as shown in Figure 3 in [17]. For ordinary non-circular sliding bearings, Pinkus and Lynn [18] solved numerical solutions of the Reynold equations and studied state load capacity and power loss by the finite difference method in 1956. After that, many researchers used different numerical techniques to study the performance of non-circular sliding bearings [17,19,20,21,22]. With reference to the ordinary non-circular sliding bearing model, the ESB model is proposed, and the lubrication characteristics of the ESB model are analyzed using ANSYS FLUENT.

The parameters and variables used herein are shown in Table 1.

### 3.2. Governing Equations

To calculate the loading capacity of the lubricating film, its pressure distribution needs to be calculated. To calculate the friction force, it is necessary to calculate the shear stress distribution. To calculate the flow rate of the fluid, it is necessary to calculate the velocity distribution. To this end, we must first establish the basic relationship between fluid stress, specific pressure, and velocity, that is, the momentum conservation equation, continuity equation and law of viscosity, which are used as the basis for solving the problem [23].

In this article, the derivation of the equations is based on the following assumptions:

(1) The lubricant flow is assumed to be isothermal flow, that is, the change in the viscosity and density of the lubricant with temperature is not considered in the study.
(2) As the Reynolds number is very low, laminar flow is assumed, and there is no vortex and turbulence.
(3) The fluid does not slip on the solid interface, that is, the velocity of the fluid particle attached to the interface is the same as the velocity of the interface. According to Westerberg et al.’s research [24,25], the grease grading NLGI 2 is completely yielded in the tiny gap at high shear rates, allowing the wall slip effect without consideration in this article.
(4) The lubricant is a non-Newtonian fluid and the relation between the shear stress and the shear rate of the lubricant is expressed by the Herschel–Bulkley model.
(5) In the direction along the thickness of the lubricating film, the pressure change is not counted because the film thickness is small compared to the diameter and width of the structure. From this hypothesis, an inference can be drawn, that is, the viscosity and density of the fluid do not change in the thickness direction.

The general mass conservation equation for incompressible as well as compressible flow is given by

(1) $$\frac{\partial \rho }{\partial t}+\nabla \cdot \left(\rho \vec{v}\right)=0$$

where *ρ* is the density of the fluid and $\vec{v}$ is the velocity vector of the fluid flow. For incompressible flow, the mass conservation equation can be simplified to

$$\nabla \cdot \left(\vec{v}\right)=0$$

equals to:

(2) $$\frac{\partial v_{x}}{\partial x}+\frac{\partial v_{y}}{\partial y}+\frac{\partial v_{z}}{\partial z}=0$$

The momentum conservation equation can be written as:

(3) $$\frac{\partial }{\partial t}\left(\rho \vec{v}\right)+\nabla \cdot \left(\rho \vec{v}\vec{v}\right)=-\nabla p+\nabla \cdot (\overset{𝄗}{\tau })+\rho \vec{g}+\vec{F}$$

where $\nabla \cdot \left(\rho \vec{v}\vec{v}\right)$ represents the fluid convection term, $\nabla \cdot (\overset{𝄗}{\tau })$ represents the viscosity term, and $\rho \vec{g}$ and $\vec{F}$ are the gravitational body force and external body forces, respectively. The stress tensor $\overset{𝄗}{\tau }$ is described by

(4) $$\overset{𝄗}{\tau }=\mu \left[\left(\nabla \vec{v}+{\left(\nabla \vec{v}\right)}^{T}\right)-\frac{2}{3}\nabla \cdot \vec{v}\overset{𝄗}{I}\right]$$

where $\overset{𝄗}{I}$ is the unit tensor and the second term on the right hand side is the effect of volume dilation.

In the coordinate system shown in Figure 2, to take into account rheological laws of lubricant, the velocity gradients (the shear strain rates) are expressed as

(5) $$\dot{\gamma }=f\left(\tau ,\mu \right)$$

In this paper, the Herschel–Bulkley model is used to express the relationship between shear stress and strain rate of grease. The standard Herschel–Bulkley model is described by:

(6) $$\tau =\tau_{0}+K{\left(\dot{\gamma }\right)}^{n}$$

where τ is the shear stress,$\;\dot{\gamma }$ is the shear strain rate, $\tau_{0}$ is the yield stress, *K* is the consistency constant of the grease, which is the plastic viscosity similar to the viscosity of the oil, and *n* is the power law constant.

A set of functions called the equivalent viscosity [26] is defined as

(7) $$\mu^{*}=\frac{\tau }{f\left(\tau ,\mu \right)}\;$$

The purpose of using equivalent viscosity is to unify the flow of Newtonian fluid and non-Newtonian fluid. Substituting Equation (5) into Equation (7) yields Equation (8) which is the viscosity law of lubricant in this article:

(8) $$\tau =\mu^{*}\dot{\gamma }$$

Equation (2), (3), (6), and (8) are combined to solve the Reynolds equation. The form of the Reynolds equation that takes into account the equations describing the non-Newtonian lubricant is

(9) $$\frac{\partial }{\partial x}\left(\frac{\rho h^{3}}{12\mu^{*}}\frac{\partial p}{\partial x}\right)+\frac{\partial }{\partial z}\left(\frac{\rho h^{3}}{12\mu^{*}}\frac{\partial p}{\partial z}\right)=\frac{\partial }{\partial x}\left(\frac{\rho Uh}{2}\right)+\frac{\partial }{\partial z}\left(\frac{\rho Wh}{2}\right)-\rho V+h\dot{\rho }$$

As W = 0 and incompressible, the formula becomes

(10) $$\frac{\partial }{\partial x}\left(\frac{h^{3}}{\mu^{*}}\frac{\partial p}{\partial x}\right)+\frac{\partial }{\partial z}\left(\frac{h^{3}}{\mu^{*}}\frac{\partial p}{\partial z}\right)=6U\frac{\partial h}{\partial x}-12V$$

where U is the velocity of the inner wall of the FS parallel to the film and V is the squeeze-film velocity. In Equation (10), the first term on the right is the hydrodynamic effect, and the second term represents the squeeze effect. When Equation (10) is applied to a stable bearing, the term of the squeeze effect can be omitted, that is V = 0.

To solve the Reynolds equation, the pressure boundary conditions should be used to determine the integration constants. There are commonly three forms of pressure boundary conditions, namely Sommerfeld boundary condition, half-Sommerfeld boundary condition, and Reynolds boundary condition. Lubricants cannot withstand continuous negative pressure, so the Sommerfeld boundary condition is not applicable to the lubrication of elliptical sliding bearings. The half-Sommerfeld boundary condition is to process the obtained pressure distribution and discard all negative pressures. The half-Sommerfeld boundary condition is simple and intuitive, and is adopted in this article. Taking into account this situation, the following boundary condition is used which applies the negative pressure constraint: p = pa, when the oil film pressure is lower than the ambient pressure [27].

**Figure 4:**
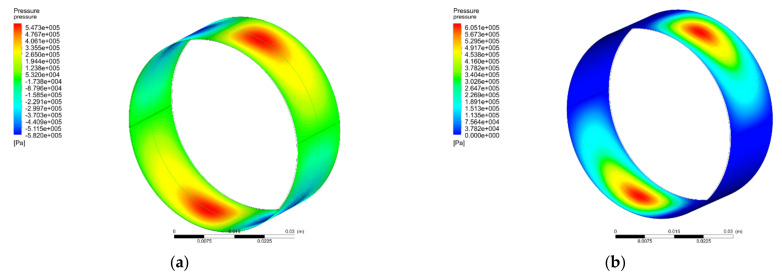
Pressure contour in Pa on the journal surface: (**a**) without applying half-Sommerfeld boundary condition, and (**b**) after applying half-Sommerfeld boundary condition when the film pressure is lower than the atmospheric pressure.

Figure 4 shows the contour diagrams of pressure distribution on the inner wall of the FS obtained without applying half-Sommerfeld boundary condition, and after applying half-Sommerfeld boundary condition when the film pressure is lower than the atmospheric pressure.

### 3.3. Numerical Model

In the present work, an ESB model for CFD analysis is proposed, using ANSYS FLUENT, to study the lubrication characteristics of the HD with NFWG lubricated with grease. The geometry of the CFD model is established as an ESB model, as shown in Figure 2, then the oil film area shown in Figure 2 is the computational domain of the simulation. The analysis type of the geometry is “3D”, and the width of the model in the z direction is 20 mm. The geometry uses structured grids to discretize the computational domain, and the model has meshed with hexahedral cells. The model is divided using the “edge sizing” method to set the number of cells. To meet grid independence and reduce calculation, 6 divisions were used across the oil film, 360 divisions were used in the circumferential direction, and 10 divisions were used in the axial direction. The total number of cells is 21,600.

The equations are solved in a steady state, taking gravity into account. The viscous model is laminar due to the low Reynolds number. The viscosity grade of the grease used in the HD herein is classified as NLGI 2, the viscosity adopts the Herschel–Bulkley model. The parameter set refers to the results given in [24] and *ρ* = 930 kg/m^3^, *K* = 20.6 Pa⋅s^n^, *n* = 0.605, *τ*_0_ = 650 Pa, and ${\dot{\gamma }}_{0}$ = 32 s^−1^. The range of rotational speed is 1000 rpm to 3000 rpm.

The operating pressure is 101,325 Pa. The boundary conditions are “pressure inlet” and “pressure outlet” with gauge pressure as zero. There are two positions with the largest oil film thickness in the circumferential direction, one side is the pressure inlet (θ = 0°) and the other side is the pressure outlet (θ = 180°). The “dynamic mesh” model is used to simulate the fluid flow since the fluid domain is changing due to motion of the boundaries. The “smoothing” mesh method is used to update grid, associated with the “remeshing” method.

In the HD, the CS is fixed and the FS rotates in the opposite direction relative to the NFWG. The rotational speed of the FS and the NFWG meet the transmission ratio. The rotational speed of the NFWG is *n*_1_ in the counterclockwise direction, and the transmission ratio is *i*, so the rotational speed of the FS is *n*_1_/*i* in the clockwise direction. According to the principle of relative motion, the NFWG boundary is modeled as a “moving wall” with a rotational speed of (*i* + 1) *n*_1_/*i* in the counterclockwise direction. The inner wall of the FS boundary is modeled as a “stationary wall”. A no-slip condition is used on the two boundaries. When the NFWG rotates at a given speed, the FS generates flowing deformation waves. Considering the principle of relative motion again, the lubricating fluid flows in the gap between the NFWG and the FS, regarded as flowing in a fixed-shaped elliptical gap with a given boundary velocity for simulation. The multiple pairs of meshing teeth between the FS and the CS provide a wide range of stable constraints for the FS, so that the FS maintains a certain deformed shape. Therefore, the interaction between the deformation of the FS and the flow of lubricating fluid is not considered.

The “pressure-based” solver is chosen for the present numerical analysis. The pressure–velocity coupling is treated using the “SIMPLE” algorithm, the “second-order upwind” discretization scheme is used for the momentum equations, and the pressure difference format is “PRESTO!”. For greater accuracy, a convergence tolerance of 10^−6^ is used for all residual terms and the number of iterations equals to 200. The mass flow of the inlet and outlet fluids are monitored during the calculation process. When the values are basically equal, the calculation will converge.

## 4. Results and Analysis

By integrating the pressure on the inner wall of the FS, the loading capacity of the model can be formulated by

(11) $$F_{p}=\iint p\;dA$$

The friction force can be calculated by integrating the shear stress on the inner wall of the FS as follows

(12) $$F_{f}=\iint \tau \;dA$$

where *A* refers to the total area of the inner wall of the FS. 

The friction factor is identified by

(13) $$f=\frac{F_{f}}{F_{p}}$$

The lubricant flow rates *Q*_0_ and *Q*_1_ at the maximum and minimum oil film thickness are given by integrating the tangential velocity of the lubricant [27]

$$Q_{0}=\iint u_{0}dA_{h_{0}}$$

$$Q_{1}=\iint u_{1}dA_{h_{1}}$$

The rate of lubricant loss due to side leakage is

(14) $$Q_{s}=Q_{0}-Q_{1}$$

**Figure 5:**
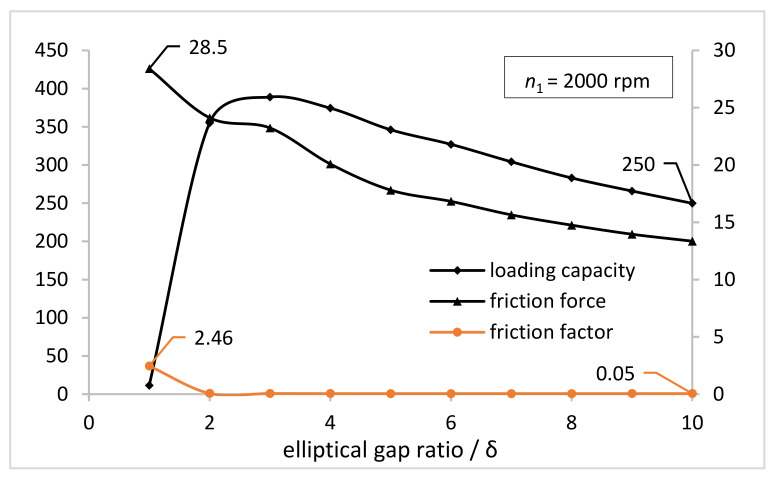
Variations of the loading capacity and friction force and friction factor on the inner wall of the FS with the elliptical gap ratio (*n*_1_ = 2000 rpm).

**Figure 6:**
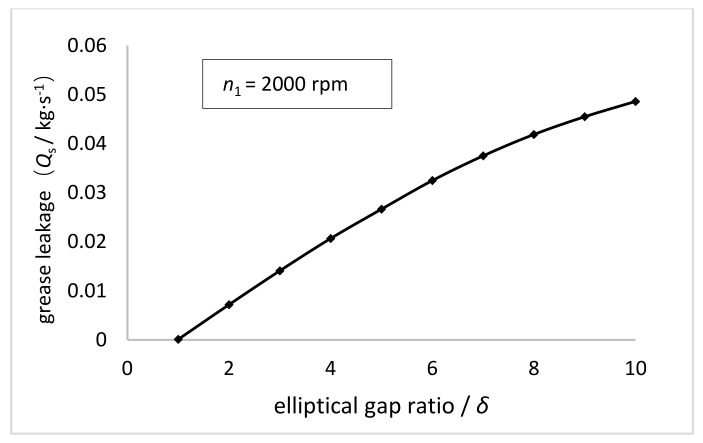
Variations of the grease leakage with the elliptical gap ratio (*n*_1_ = 2000 rpm).

As shown in Figure 5, when the rotational speed of the NFWG is 2000 rpm, the variation curves of loading capacity, friction force, and friction factor on the inner wall of the FS are given as the increase of the elliptical gap ratio between the NFWG and the FS which is identified in Table 1. The elliptical gap ratio as an indicator is utilized to design the NFWG. Figure 6 shows the variation curve of the leakage of lubricating grease from both sides of the gap as the increase of the elliptical gap ratio. It can be seen that as the elliptical gap ratio increases, the leakage of grease also increases monotonically.

Figure 5 shows that the loading capacity first increases rapidly and then decreases with the increasing elliptical gap ratio, and the turning point is 3. The loading capacity is positively related to the surface area and the pressure of the inner wall of the FS. The surface area of the inner wall of the FS is constant whereas the pressure on the inner wall of the FS first increases and then decreases as the elliptical gap ratio increases. The trend of pressure is related to the hydrodynamic effect generated in the gap between the NFWG and the FS. Hydrodynamic effect is based on the geometric shape and relative movement of the friction surface, with the help of the dynamic action of the viscous fluid to generate the hydrodynamic pressure to support the external load. When the hydrodynamic pressure is balanced with the external load, a stable oil film is formed to realize hydrodynamic lubrication. There are several types of hydrodynamic lubrication according to the difference in formation method. The method of forming a hydrodynamic oil film by the convergent gap between two surfaces is the most common type in hydrodynamic lubrication problems. Regarding the ESB model herein, when the NFWG rotates in the FS, the convergent gap will be produced between the FS and the NFWG, and a hydrodynamic oil film can also be formed in the gap. Moreover, the hydrodynamic pressure increases as well as loading capacity as the elliptical gap ratio increases. However, as the elliptical gap ratio continues to increase, the excessive leakage of the lubricant does serious harm to the formation of hydrodynamic pressure resulting in the decrease of loading capacity.

Figure 5 also shows that the friction force on the inner wall of the FS presents a downward trend, but the decline rate gradually decreases as the increase of elliptical gap ratio. The friction force is the integral of the surface shear stress. According to the definition of shear stress, the shear stress decreases as the gap increases, resulting in a decrease in friction force. The curve correlation between the friction factor defined by the ratio of friction force and loading capacity on the inner wall of the FS and the elliptical gap ratio is also plotted in Figure 5. As the elliptical gap ratio increases to 3, the friction factor drops rapidly from 2.5 to 0.05, and then the friction factor is basically unchanged. According to the definition given in Table 1, when the elliptical gap ratio is 1, the gap between the NFWG and the inner wall of the FS is unchanged in the circumferential direction. At this time, there is no convergent gap, and there is no necessary condition for the formation of hydrodynamic lubrication, resulting in poor lubrication performance. When the elliptical gap ratio increases to 2, the convergent gap is formed between the NFWG and the inner wall of the FS, which produces a hydrodynamic lubrication effect. This is the reason why the friction factor drops sharply when the elliptical gap ratio changes from 1 to 3. This impact is also reflected in the dramatic increase of loading capacity.

Considering comprehensively, when the elliptical gap ratio is 3, the lubrication performance of the model is optimal, which is utilized to optimize the NFWG hereinafter.

**Figure 7:**
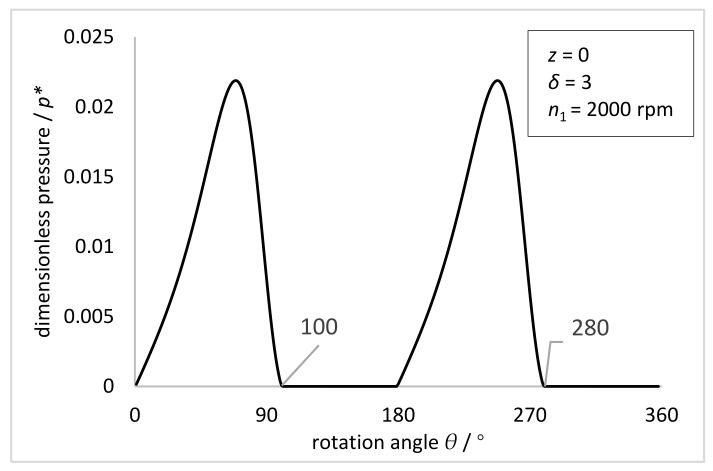
The dimensionless pressure distribution on the inner wall of the FS at z = 0 with the rotation angle θ (*z* = 0, *δ* = 3, *n*_1_ = 2000 rpm).

Figure 7 shows the dimensionless pressure distribution on the inner wall of the FS when the elliptical gap ratio is 3 and the rotational speed of the NFWG is 2000 rpm. The pressure presents a periodic distribution, and there are two periods between 0 and 2π. The negative pressure is always being zero because of setting the pressure zero when the film pressure is lower than the atmospheric pressure.

**Figure 8:**
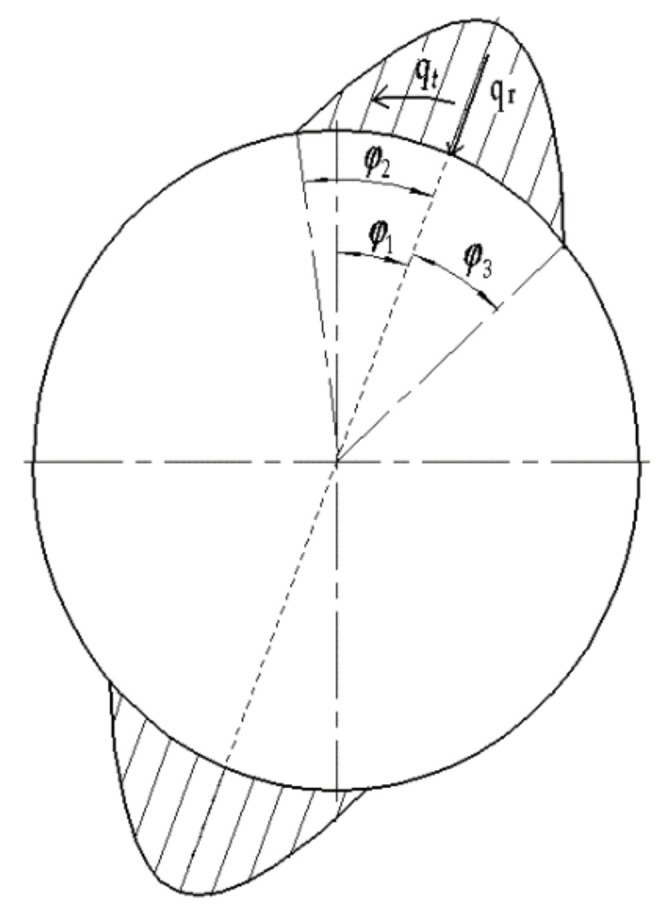
External load distribution of FS of the HD with a conventional FWG.

Figure 8 shows the experimental distribution of meshing force on the FS of the HD with a conventional FWG given in [28].

The external load distribution of the FS in the HD with a conventional FWG can be expressed as

(15) $$\left\{\begin{array}{c} {\bar{q}}_{t}={\bar{q}}_{t,max}\cos\left[\pi \left(\varphi -\varphi_{1}\right)/2\varphi_{2}\right]\\ {\bar{q}}_{r}={\bar{q}}_{t}\tan\alpha \end{array}\right.$$

where *q_t_* is the circumferential uniform load, *q_r_* is the radial uniform load, *α* is the tooth profile angle, *φ*_1_ is the deflection angle of the load, which represents the deflection angle of the symmetry axis of the distributed load relative to the major axis of the wave generator, and *φ*_2_ is the left distribution angle of load, *φ*_3_ is the right distribution angle of the load [28].

The FWG can only withstand positive pressure from FS. Therefore, the load on the wave generator can be written as

(16) $${\bar{q}}_{r}={\bar{q}}_{r}={\bar{q}}_{t,max}\cos\left[\pi \left(\varphi -\varphi_{1}\right)/2\varphi_{2}\right]\tan\alpha$$

In this paper, *φ*_2_ = *φ*_3_ = π/8, *φ*_1_ = π/8. The load of the FS can be simplified to

(17) $${\bar{q}}_{r}={\bar{q}}_{r,max}\cos\left[\frac{\pi \left(\varphi -\frac{\pi }{8}\right)}{2\varphi_{2}}\right]$$

(18) $${\bar{q}}_{r,max}={\bar{q}}_{t,max}\tan\alpha =\frac{\pi T_{2}}{2\varphi_{2}d_{g}{}^{2}b_{w}}\tan\alpha$$

where *p* is the gear tooth pitch, *d_g_* is the diameter of the pitch circle, *b_w_* is the working width of the gear ring of the FS, *T*_2_ is the torque, and *T*_2_ = 18 N·m, *α* = 23°, *d_g_* = 50 mm, *b_w_* = 20 mm.

The traditional FWG converts the sliding friction into rolling friction through the flexible bearing, thereby reducing the wear of the inner wall of the FS and improving the lubrication performance. Compared with the traditional FWG with flexible bearing, the NFWG without flexible bearing improves lubrication performance with hydrodynamic lubrication. The moving surfaces are separated by hydrodynamic oil film, thereby reducing friction and wear. The method of hydrodynamic lubrication is simple and effective and has excellent lubrication characteristics. The disadvantage is that since the hydrodynamic lubricant film must be formed during movement, the structure has a larger friction and a higher starting torque in the initial stage of rotation.

**Figure 9:**
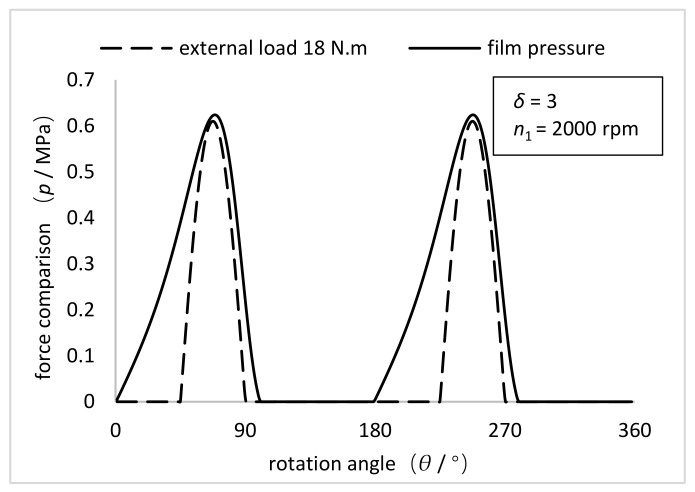
Comparison between the simulation results of pressure distribution of the oil film on the inner wall of the FS with the NFWG and the experimental results of external load distribution of the FS with the conventional FWG under the torque of 18 N·m (*δ* = 3, *n*_1_ = 2000 rpm).

As shown in Figure 9, the comparison between the simulation results of pressure distribution of the oil film on the inner wall of the FS with the NFWG and the experimental results of external load distribution of the FS with the conventional FWG under the torque of 18 N·m is given. For HD, the stress in the FS depends on the load transmitted by the transmission, as well as the size of the meshing area and the distribution of loads in the meshing area. The radial load in meshing and the reaction force of the FWG are equal in magnitude but opposite in direction. Therefore, the pressure distribution represented by the dotted line in Figure 9 is equivalent to the force of the FWG on the entire circumference. The pressure distribution represented by the solid line in Figure 9 is the pressure distribution on the inner wall of the FS under the same size condition, which is equivalent to the force of the NFWG. Through comparison, it can be seen intuitively that the solid line wraps the dotted line, which proves that the novel HD with the NFWG can withstand a torque of 18 N·m under current condition. It verifies that the NFWG with optimal elliptical gap ratio in the HD can provide load and transmission under hydrodynamic lubrication with small friction factor, thereby reducing the wear of the FS and improving the lubrication performance.

**Figure 10:**
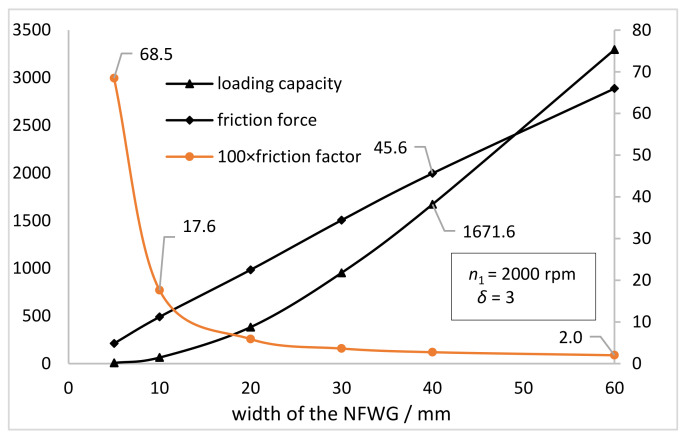
Variations of the loading capacity and friction force and friction factor on the inner wall of the FS with the width of the NFWG (*n*_1_ = 2000 rpm, *δ* = 3).

Figure 10 shows the variation curve of the loading capacity and friction force and friction factor on the inner wall of the FS as the increase of the width of the NFWG. It can be seen that loading capacity and friction force increase monotonously as the increase of the width of the NFWG from 10 mm to 60 mm. This is because the larger the width, the larger the surface area and the greater the friction force and the oil film pressure. The friction factor first decreases sharply and then tends to be flat, decreasing from 0.685 to 0.02. The sharp drop in the friction factor is due to the reduction of lubricant leakage and the formation of hydrodynamic lubrication between the NFWG and the inner wall of the FS. It can be concluded that the width of the NFWG affects greatly the friction performance of the model, but for a given harmonic drive unit, that is, the inner diameter of the FS, the width of the NFWG is also given based on the design specifications. In this research, the width of the NFWG is equal to the working width of the FS.

**Figure 11:**
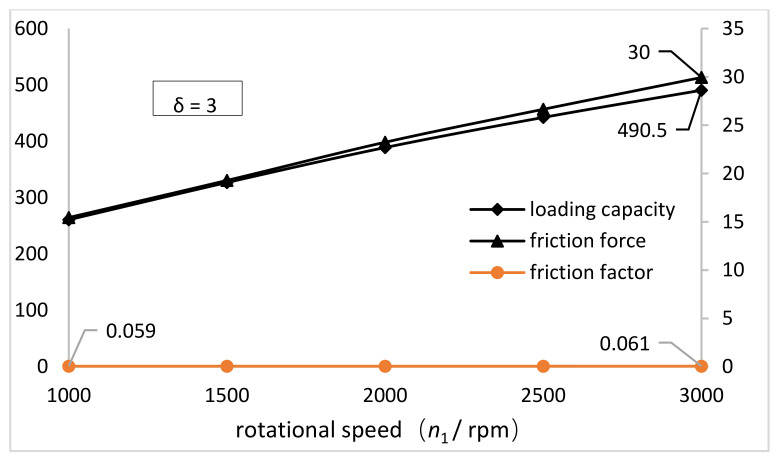
Variations of the loading capacity and friction force and friction factor on the inner wall of the FS with the rotational speed of the NFWG (*δ* = 3).

Figure 11 shows the variation curve of the loading capacity and friction force on the inner wall of the FS as the increase of the rotational speed of the NFWG. It can be seen that loading capacity and friction force increase monotonously as the increase of rotational speed from 1000 rpm to 3000 rpm. This is because the higher the speed, the more lubricant passing through the surface per unit time, the greater the friction force and the oil film pressure. The loading capacity increased by about 230 N, and the increase rate was about 88%. Compared with the loading capacity, the friction force increased by about 14 N and the increase rate was about 93%. The synchronous change of the loading capacity and the friction force is responsible for little fluctuation around 0.06 of the friction factor. It can be concluded that the rotational speed of the NFWG has a limited effect on the friction performance of the model under conditions herein. As shown in Figure 11, the friction factor is 0.058 when the elliptical gap ratio is 3, which indicates there is a good lubrication condition between the NFWG and the FS. Under this lubrication condition, the practical design of the NFWG is given.

## 5. NFWG Practical Design

**Figure 12:**
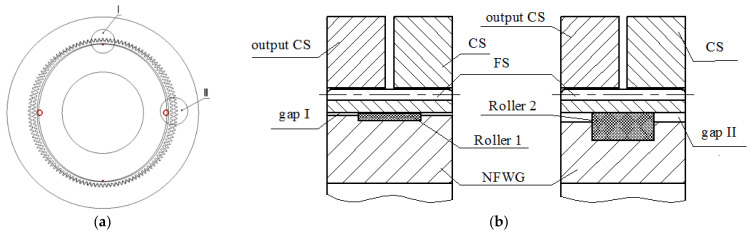
(**a**) Design diagram. (**b**) partial enlarged schematic diagram at major axis of the NFWG I and minor axis of the NFWG II.

According to the analysis of simulation results, the practical design of the NFWG in pancake harmonic drive is carried out. The NFWG is designed under the optimal elliptical gap ratio, and the gap between the NFWG and the FS is filled with grease. Figure 12a shows the design diagram. Figure 12b shows partially enlarged schematic diagrams at major axis of the NFWG I and at minor axis of the NFWG II. To maintain the gap between the NFWG and the FS and to support the deformation of the FS at rest, the design of embedding cylindrical or spherical rollers of the NFWG is proposed as shown in Figure 12b. The gap between the FS and the NFWG at the minor axis is 3 times larger than that at the major axis, so the rollers at the minor axis are 3 times larger than that at the major axis.

## 6. Discussion

From the above analysis of simulation results, the design of the NFWG affects the lubrication performance significantly and the rotational speed of the NFWG has limited effect on lubrication performance. Moreover, when the elliptical gap ratio is 3, the lubrication performance of the model is optimal, which is utilized to optimize the NFWG. The comparison between the simulation results of pressure distribution on the inner wall of the FS with the NFWG and the experimental results of external load distribution of the FS with the conventional FWG under the torque of 18 N·m is given, which verifies that the rationality and practicability of the design of the NFWG in HD. According to the analysis, the practical design of the NFWG in pancake harmonic drive is given. The effect of the rollers of the NFWG is to maintain the gap between the NFWG and the FS and to support the deformation of the FS at rest. The rollers at the minor axis are 3 times larger than that at the major axis of the NFWG. The influence of rollers on lubrication characteristics of the NFWG will be studied in future work.

## 7. Conclusions

According to the simulation results, the effect of the elliptical gap ratio, width, and the rotational speed of the NFWG on the loading capacity, friction force, grease leakage, and friction factor of the model is analyzed. With the increasing elliptical gap ratio, the loading capacity on the inner wall of the FS first increases rapidly and then decreases, and the turning point is 3. The friction force presents a downward trend, but the decline rate gradually decreases. The leakage of grease increases monotonically. As the elliptical gap ratio increasing to 3, the friction factor drops rapidly from 2.5 to 0.05, and then the friction factor is basically unchanged. When the elliptical gap ratio is 3, the lubrication performance of the model is optimal, which is utilized in the design of the NFWG. As the width of the NFWG increases from 10 mm to 60 mm, the loading capacity and friction force increase monotonously. The friction factor first decreases sharply and then tends to be flat, decreasing from 0.685 to 0.02. As the rotational speed increases from 1000 rpm to 3000 rpm, the loading capacity and friction force increase monotonously. The friction factor has little fluctuation around 0.06.

By comparing the simulation results of pressure distribution of the oil film on the inner wall of the FS with the NFWG with the experimental results of external load distribution of the FS with the conventional FWG under the torque of 18 N·m, it verifies that the NFWG with optimal elliptical gap ratio in HD can provide load and transmission under hydrodynamic lubrication with small friction factor, thereby reducing the wear of the FS and improving the lubrication performance of the NFWG. Then, the practical design of the NFWG in pancake harmonic drive is given. The NFWG of HD has the characteristics of compact structure, and low noise when working.

The work about analysis of lubrication characteristics of the NFWG of the HD and design of the NFWG carried out here provides a basis for the structural design and optimization of the FWG without flexible bearing of the HD. To further guide design of the FWG without flexible bearing, the experimental analysis of the NFWG will be focused on in our future work.

## Figures and Tables

**Figure 3:**
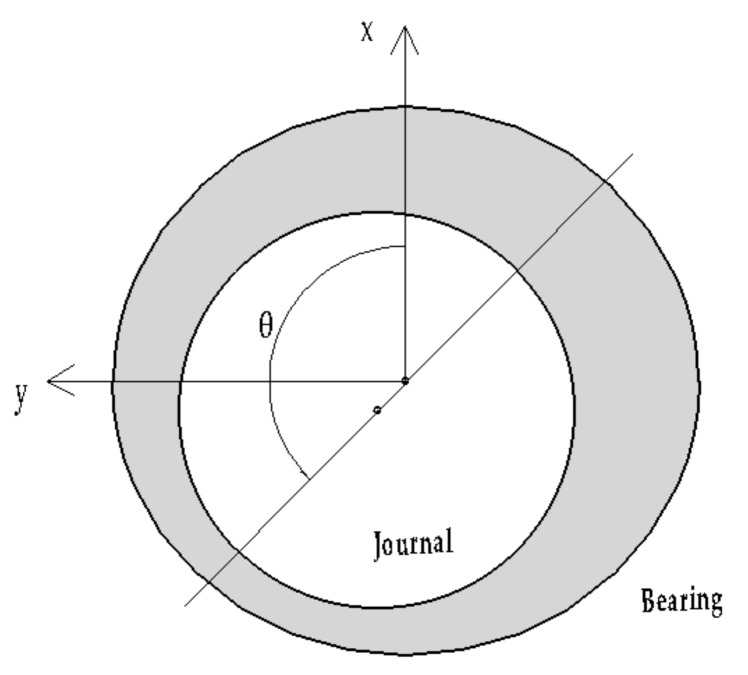
Ordinary non-circular sliding bearing.

**Table 1:** Parameters and variables.

Symbols and Definitions			
*a*_1_/*b*_1_	Radius of the major and minor axis of the inner wall of the FS, mm		
*r*	Radius of the inner wall of the FS, mm	*b*_w_	Working width of the FS, mm
*a*_2_/*b*_2_	Radius of the major and minor axis of NFWG, mm	*L*	Width of the NFWG, mm
*h*_max_/*h*_min_	Maximum and minimum oil film thickness,	*h*	Oil film thickness, mm
*ψ*	Top gap, *ψ = a*_1_ − *a*_2_, mm	*n*	Power law constant
*C*	Side gap, *C = b*_1_ − *b*_2_, mm	*K*	Consistency constant of grease, Pa·s^n^
*δ*	Elliptical gap ratio, *δ* = *C*/*ψ*	*τ*_0_	Yield stress, Pa
*p*	Pressure, Pa	*τ*	Shear stress, Pa
p*	Dimensionless pressure, p* = (*p* − *p*_a_) *C^2^*/(*μωr^2^*)	*μ*	Viscosity, Pa·s
*p*_a_	Atmospheric pressure, 101,325 Pa	${\dot{\gamma }}_{0}$	Critical shear strain rate, s^−1^
*n*_1_	Rotational speed of the NFWG, rpm	$\dot{\gamma }$	Shear strain rate, s^−1^
*ω*	Rotational angular velocity of the NFWG, *ω = πn*_1_/30, rad·s^−1^	*F*_p_	Loading capacity, N
*ρ*	Density of lubricant, kg·m^−3^	*F*_f_	Friction force, N
*μ*_0_	Yield viscosity, Pa·s	*f*	Friction factor
*Re*	Reynolds number, *Re = ρωrC*/*μ*_0_	*Q*_s_	Grease leakage, m^3^·s^−1^
*i*	Transmission ratio of HD		

## Data Availability

The data presented in this study are available on request from the corresponding author.
